# Long-term treatment outcomes of ritonavir-boosted lopinavir monotherapy among HIV-infected patients who experienced NRTI and NNRTI failure

**DOI:** 10.1186/1742-6405-9-8

**Published:** 2012-03-13

**Authors:** Weerawat Manosuthi, Supeda Thongyen, Samruay Nilkamhang, Sukanya Manosuthi, Somnuek Sungkanuparph

**Affiliations:** 1 Bamrasnaradura Infectious Diseases Institute, Ministry of Public Health, Nonthaburi 11000, Thailand; 2 Faculty of Medicine Ramathibodi Hospital, Mahidol University, Bangkok, Thailand

**Keywords:** HIV, Lopinavir, Monotherapy, Lamivudine, Resistance, Thailand

## Abstract

**Background:**

We continue the previously described prospective cohort study of ritonovir-boosted lopinavir (LPV/r) monotherapy for second-line therapy in HIV-infected patients with prior failure and extensive resistance to nucleoside reverse transcriptase inhibitors (NRTIs) and non-nucleoside reverse transcriptase inhibitors (NNRTIs), with the objective being to determine the three-year treatment responses.

**Findings:**

There were 40 patients with a mean ± SD age of 37 ± 8 years. Median (IQR) baseline CD4 was 123 (37-245) cells/mm^3 ^and median (IQR) HIV-1 RNA was 55,800 (9,670-100,000) copies/mL. All patients received twice daily LPV/r 400/100 mg and recycled lamivudine 150 mg. By intend-to-treat analysis at 144 weeks, 26 (65%) and 22 (56%) patients achieved HIV-1 RNA at < 400 and < 50 copies/mL, respectively. In as-treated analysis, the corresponding rates were 26 of 28 (93%) and 22 of 28 (78%), respectively. Low-level viral rebound (HIV-1 RNA 50-400 copies/mL) was found in 6 (15%), 6 (15%), and 4 (10%) patients at week 48, 96 and week 144, respectively. Medians CD4 at week 48, 96, and 144 were 351, 481, and 584 cells/mm^3 ^and significantly changed from baseline (all, *P *< 0.05). There were increments of mean triglycerides at 48 weeks and 144 weeks from baseline (*P *< 0.05). No major protease resistance-associated mutations emerged after virologic failure.

**Conclusion:**

LPV/r monotherapy with recycled lamivudine can maintain long-term virologic suppression in a relatively small proportion of patients failing NNRTI-based regimen and having limit option for active NRTI. More antiretroviral classes are needed be accessible in resource-limited countries.

## Findings

There are many concerns raised regarding boosted protease inhibitor monotherapy in HIV treatment include this strategic treatment may not be as effective as other combined antiretroviral therapies (ART), high rate of low-level viremia and may lead to developing treatment failure, and a higher level of adherence is required than with the use of standard combined ART [1]. In addition, there is an important concern about the ability of monotherapy to penetrate viral reservoirs and prevent viral replication in sanctuary sites, such as genital tract and central nervous system. Studies evaluating ritonavir-boosted protease inhibitor monotherapy that derived from the western countries have been studied in three patient settings including initial treatment, induction-maintenance, and simplification monotherapy after patients have been virologically suppressed - all are in patients without treatment failure [2]. However, data regarding durability of this strategic treatment is still scanty, owing to supporting by only relatively short-term data. On the other hand, HIV is often resistant to most existing nucleoside reverse transcriptase inhibitors (NRTIs) and non- nucleoside reverse transcriptase inhibitors (NNRTIs) among patients who have failed with the first regimen in resource-constrained settings, secondary to the delayed detection of treatment failure. Therefore, constructing the next antiretroviral regimens that combined three fully active drugs for HIV-infected patients with prior failure and extensive resistance to NRTIs and NNRTIs in such setting is often impossible. Therefore, we conducted a prospective cohort study of ritonavir-boosted lopinavir monotherapy for the second-line therapy in HIV-1 infected patients who failed antiretroviral regimens containing NRTIs and NNRTI as previously described [3]. In the present analysis, we continued the prospective cohort study with the objectives to determine the three-year virologic and immunologic responses, and lipid derangements.

The present study was designed as a prospective cohort study involving 40 HIV-1 infected patients who were diagnosed virologic failure at the Bamrasnaradura Infectious Diseases Institute, Ministry of Public Health, Thailand. Virologic failure was defined as having viral load > 1,000 copies/mL after 6 months of treatment or a rebound of viral load to > 1,000 copies/mL in any duration after undetectable viral load. Discontinuation of lopinavir/ritonavir due to any reason was considered to be a treatment failure, i.e. plasma HIV-1 RNA > 1,000 copies/mL. Inclusion criteria were as follows: (1) HIV-1 infected patients > 18 years of age, (2) failed NNRTI-based ART with M184V, thymidine analogue mutations (TAMs) and NNRTI-associated mutations, and (3) had plasma HIV-1 RNA > 1,000 copies/mL. The patients were excluded if they had a history of exposure to protease inhibitor or receipt a medication that has drug-drug interactions with lopinavir or ritonavir. Ritonavir-boosted lopinavir in soft gel formulation at 400/100 mg and lamivudine at 150 mg were given twice daily. Ritonavir-boosted lopinavir soft gel formulation was changed to tablet formulation after 48 weeks of treatment. CD4 cell counts (flow cytometry), plasma HIV-1 RNA (Roche Amplicor, version 1.5), and lipid profiles were measured every 24 weeks. Medication adherence was assessed by pill count. All analyses were performed using SPSS software, version 15.0 (SPSS Inc., Chicago, IL, USA). This study was reviewed and approved by ethical committee for research in human subjects of the Department of Diseases Control, Ministry of Public Health and by the institutional review board.

As initial enrollment, there were 40 patients with a mean ± SD age of 37 ± 8 years and 70% were males. Median (IQR) baseline CD4 cell count was 123 (37-245) cells/mm^3 ^and median (IQR) plasma HIV-1 RNA was 55,800 (9,670-100,000) copies/mL. Mean ± SD baseline total cholesterol was 165 ± 42 mg/dL and mean ± SD triglycerides was 172 ± 117 mg/dL. The frequencies of each thymidine analogue associated mutation (TAMs) were as follows: D67N, 17 (43%), T215FY, 16 (40%), M41L, 8 (20%), K65R, 6 (15%), L210W, 6 (15%), and K219Q, 2 (5%). M184V, Q151M and L74V were found in 40 (100%), 7 (18%) and 2 (5%), respectively. The prevalence of patients with ≥1 major mutation conferring drug resistance to NNRTIs was 100%.

**Figure 1:**
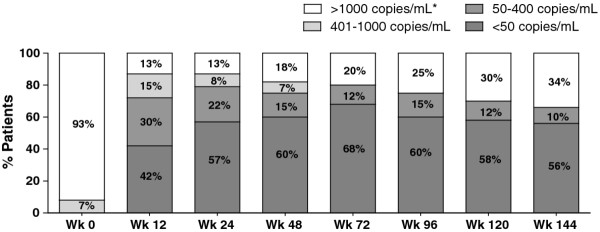
**Percentage of patients who had different stratum of plasma HIV-1 RNA at each follow-up visit of treatment by intend-to-treat analysis**.*Discontinuation of lopinavir/ritonavir due to any reason was considered to be plasma HIV-1 RNA > 1,000 copies/mL.

Figure 1 displayed the proportion of patients who had different stratum of plasma HIV-1 RNA at each follow-up visit by intend-to-treat analysis. At 144 weeks, 26 (65%) and 22 (56%) patients had achieved plasma HIV-1 RNA at < 400 and < 50 copies/mL, respectively. In as-treated analysis, the corresponding rates were 26 of 28 (93%) and 22 of 28 (78%), respectively. Low-level viral rebound (HIV-1 RNA 50-400 copies/mL) was found in 6 (15%), 6 (15%), and 4 (10%) patients at week 48, 96 and week 144, respectively. Medians CD4 cell count at weeks 48, 96, and 144 were 351, 481, and 584 cells/mm^3 ^and significantly changed from baseline (all, *P *< 0.05). Compared measures to baseline values, there were increments of mean triglycerides at 48 weeks and 144 weeks from baseline (172 mg/dL vs. 338 mg/dL and 320 mg/dL, *P *< 0.05). LDL-cholesterol was not different (*P *> 0.05). No major protease resistance-associated mutations emerged after virologic failure. Three patients died over the study period. The attributed causes of death were cryptococcal meningitis (1 patient), sepsis (1), and hepatits B virus-associated cirrhosis with fulminant hepatitis (1). Over the study period, 36, 32, and 28 patients continued to follow-up at weeks 48, 96, and 144 weeks.

A recent systematic review of lopinavir/ritonavir monotherapy demonstrated that overall response rate is not as effective as either continuing combined ART or giving ART at the beginning with relatively marginal significant magnitude, i.e. 95% confidence interval for this determination was 1.02 to 2.13 [2]. Of note, this strategic treatment appears most successful when patients are simplified from suppressive ART regimens for a period of time rather than starting monotherapy at the beginning [2]. Thus, this outcome of lopinavir/ritonavir monotherapy among ART-naïve patients may imply to our patients who experienced NRTI and NNRTI treatment but were protease inhibitor-naïve and had high plasma viral load at the beginning. Our data indicate that the durability of ritonavir-boosted lopinavir monotherapy combined with recycled lamivudine regarding virologic suppression of patients who were failing NNRTI-based regimens with M184V, TAMs and NNRTI mutations is somewhat unfavorable. A recent preliminary data of a randomized, controlled, trail demonstrated that patients who failed NNRTI-based ART regimens and who were protease inhibitor-naive, ritonavir-boosted lopinavir monotherapy and ritonavir-boosted lopinavir combined with tenofovir/emtricitabine regimen had similar proportions of patients with plasma HIV-1 RNA < 400 copies/mL, but not for < 50 copies/mL [4]. It is consistent with the present study showing a high proportion of patients with persistent viremia throughout the three-year follow-up period. One possible explanation is the lack of viral suppression in some compartments, such as genital secretion and cerebrospinal fluid [5]. Another explanation is an alternative pathway of protease inhibitor resistance facilitated by the absence of the NRTI drugs, different HIV subtypes influence on polymorphisms, and a non-adherence issue [6,7]. However, HIV resistant strains may be present at levels below the limit of detection of the test. One of our patients with unsuppressed viral load throughout the study period had compliance of less than 80%. Nonetheless, emerging protease resistance-associated mutation in patients who developed virologic failure with is rare in the previous reports [8,9]. In terms of immunologic outcome, the present study reveals that ritonavir-boosted lopinavir showed a great performance on the immunological response after three years of treatment.

Another concern is that the efficacy in reservoirs is still uncertain as aforementioned. Previous data showed that most of the patients had undetectable HIV-1 RNA in semen and vaginal compartment with ritonavir-boosted lopinavir monotherapy [10]. Nonetheless, Gutmann and colleagues showed that elevated viral load in cerebrospinal fluid and abnormal neurological symptoms were found in the patients who had virologic rebound after simplified maintenance with ritonavir-boosted lopinavir monotherapy [11]. Since the central nervous system penetration-effectiveness score is lower for boosted protease inhibitor monotherapy than for standard combined ART, it has been postulated that boosted protease inhibitor monotherapy might have a higher risk for virological failure in the central nervous system and it would contribute to further neurocognitive impairment [12,13].

This study provides evidence that long-term virologic control is possible in the patients failing NNRTI-based regimen and having limited options for active NRTI with receiving ritonavir-boosted lopinavir monotherapy and recycled lamivudine. However, this success is in a relatively small proportion of patients. Ritonavir-boosted lopinavir monotherapy should be prescribed with caution as a second-line option, especially in settings where close viral load monitoring is not available. As a consequence, more antiretroviral classes to assure the potency of the regimen are needed to be accessible in many resource-constrained settings. A further larger study is required to assess the risk and benefit of this proposed strategic treatment.

## Competing interests

The authors declare that they have no competing interests.

## Authors' contributions

WM participated in the design of the study, statistical analysis and draft the manuscript. ST, SN, SM, and SS participated in the design of the study and draft the manuscript. All authors read and approved the final manuscript.
